# The genomes of three stocks comprising the most widely utilized live sporozoite *Theileria parva* vaccine exhibit very different degrees and patterns of sequence divergence

**DOI:** 10.1186/s12864-015-1910-9

**Published:** 2015-09-24

**Authors:** Martin Norling, Richard P. Bishop, Roger Pelle, Weihong Qi, Sonal Henson, Elliott F. Drábek, Kyle Tretina, David Odongo, Stephen Mwaura, Thomas Njoroge, Erik Bongcam-Rudloff, Claudia A. Daubenberger, Joana C. Silva

**Affiliations:** SLU Global Bioinformatics Centre, Department of Animal Breeding and Genetics (HGEN), Swedish University of Agricultural Sciences (SLU), Uppsala, Sweden; International Livestock Research Institute, Nairobi, Kenya; Functional Genomics Centre, ETH/UZH, Zürich, Switzerland; Institute for Genome Sciences, University of Maryland School of Medicine, Baltimore, USA; School of Biological Sciences, The University of Nairobi, Nairobi, Kenya; Swiss Tropical and Public Health Institute, Basel, Switzerland; University of Basel, Basel, Switzerland; Department of Microbiology and Immunology, University of Maryland School of Medicine, Baltimore, USA

**Keywords:** *Theileria parva*, East coast fever, Live vaccine, Muguga Cocktail, Protozoan parasite, Genome sequence, Single nucleotide polymorphisms, Comparative genomics, Vaccinology

## Abstract

**Background:**

There are no commercially available vaccines against human protozoan parasitic diseases, despite the success of vaccination-induced long-term protection against infectious diseases. East Coast fever, caused by the protist *Theileria parva*, kills one million cattle each year in sub-Saharan Africa, and contributes significantly to hunger and poverty in the region. A highly effective, live, multi-isolate vaccine against *T. parva* exists, but its component isolates have not been characterized. Here we sequence and compare the three component *T. parva* stocks within this vaccine, the Muguga Cocktail, namely Muguga, Kiambu5 and Serengeti-transformed, aiming to identify genomic features that contribute to vaccine efficacy.

**Results:**

We find that Serengeti-transformed, originally isolated from the wildlife carrier, the African Cape buffalo, is remarkably and unexpectedly similar to the Muguga isolate. The 420 detectable non-synonymous SNPs were distributed among only 53 genes, primarily subtelomeric antigens and antigenic families. The Kiambu5 isolate is considerably more divergent, with close to 40,000 SNPs relative to Muguga, including >8,500 non-synonymous mutations distributed among >1,700 (42.5 %) of the predicted genes. These genetic markers of the component stocks can be used to characterize the composition of new batches of the Muguga Cocktail.

**Conclusions:**

Differences among these three isolates, while extensive, represent only a small proportion of the genetic variation in the entire species. Given the efficacy of the Muguga Cocktail in inducing long-lasting protection against infections in the field, our results suggest that whole-organism vaccines against parasitic diseases can be highly efficacious despite considerable genome-wide differences relative to the isolates against which they protect.

**Electronic supplementary material:**

The online version of this article (doi:10.1186/s12864-015-1910-9) contains supplementary material, which is available to authorized users.

## Background

*Theileria parva* is an intracellular tick-transmitted protozoan parasite native to eastern, central, and southern Africa, and the causative agent of East Coast fever (ECF) in cattle. The vectors are ticks within the genus *Rhipicephalus,* mainly *R. appendiculatus,* and the primary mammalian host is the African Cape buffalo (*Syncerus caffer*). While the African buffalo is an asymptomatic carrier of *T. parva*, cattle are evolutionarily recent hosts and typically succumb rapidly to the disease [1]. The parasite, which is transmitted as a sporozoite during tick feeding, invades lymphocytes of buffalo and cattle. In infected lymphocytes, the parasite divides in synchrony with the host cells while inducing uncontrolled proliferation and ‘immortalization’ of the host cells. Later in the course of the infection, *T. parva* undergoes merogony resulting in lysis of transformed host cells, and finally the differentiation into the piroplasm stage. Development of the piroplasm stage occurs in red blood cells, which are ingested during tick feeding, completing the developmental cycle. Within the tick, the parasite undergoes sexual reproduction in the gut and differentiates through several stages ultimately resulting in the generation of sporozoites in the tick salivary gland, following meiosis (reviewed in [2]). Clinically, ECF is characterized by fever, generalized disease of the lymph nodes and a reduction in the number of white blood cells. Susceptible *Bos taurus* animals usually die within three to four weeks as a result of widespread lympho-cytolysis in the lymphoid tissues and pulmonary oedema associated with invasion of the lung lymph node tissue by parasitized lymphocytes [1].

A method of immunization has been established to control development of clinical ECF in cattle, based on inoculation with a preparation of live *Theileria* sporozoites extracted from ground-up, infected whole ticks [3]. A potentially fatal dose of sporozoites is inoculated subcutaneously to initiate an infection, which is then controlled by concomitant injection of a long-acting formulation of oxytetracycline. This approach is known as “Infection and Treatment Method” or ITM (reviewed in [4, 5]). Long-lasting, heterologous protection is obtained with this immunization method using the “Muguga Cocktail”, a formulation containing three stocks of *T. parva* – Muguga, Kiambu5, and Serengeti-transformed [4]. In the absence of further challenge, the ECF immunity induced by ITM vaccination lasts for at least 43 months [6], but since febrile reactions to challenge increase with time since last exposure it is believed that, with regular natural exposure to the parasite, this immunity may be maintained over a longer period of time [6].

Deployment of this vaccine has occurred primarily in the Tanzanian Maasai pastoralist sector, with approximately one million calves vaccinated to date, but it has also been used on a smaller scale in pilot projects in the dairy sector in Uganda and elsewhere in East Africa. The success of vaccination in Tanzania has stimulated regional demand for ITM including in Southern Sudan and Kenya [7]. One of the major drawbacks of ITM is that, if carried out incorrectly, the immunization procedure can lead to severe ECF-like symptoms. This problem has recently been considerably reduced by using higher doses of oxytetracycline [5].

Challenges associated with scaling up delivery of ITM are the use of live parasites, requiring a secured liquid nitrogen supply and an uninterrupted cold chain for delivery to the farmer, and the high production cost of the three independent stocks that are combined to produce the *T. parva* Muguga Cocktail. A further issue is that production of each new vaccine batch requires infection of cattle followed by application of ticks, during which genetic recombination may occur, and finally the combination of material from ground ticks, each carrying a variable amount of one of the three vaccine stocks [8]. Therefore, the composition of each batch of immunizing stabilate is likely to differ, leading to a requirement for molecular characterization of component stocks, as part of quality control. Lastly, as perhaps most significant of all, is the fact that upon ITM vaccination cattle do not clear the parasite, but instead become carriers with low-level parasitemia. Therefore, when Muguga Cocktail-based ITM vaccination is first introduced into new geographic regions, the risk of it failing to induce cross-protection against the locally circulating *T. parva* population, and the introduction of novel *T. parva* genotypes from the vaccine preparation into these parasite populations [9], necessitates close monitoring of vaccinated cattle. The remarkable success of ITM in field settings, together with the challenges highlighted above, have led to a renewed interest in identifying the molecular basis of this vaccine’s broad efficacy. In addition to improving ITM, this knowledge could also be applied to leverage development of an efficacious subunit vaccine that avoids the drawbacks of live immunization.

The advent of next generation sequencing has made determination of complete genome sequences of *T. parva* cost-effective [8]. In this study, we have sequenced the genome of the *T. parva* Muguga Cocktail vaccine component stocks Kiambu5 and Serengeti-transformed. We have also re-sequenced a clonal parasite, Muguga clone 2 (hereafter Muguga2), derived from the *T. parva* Muguga stock, which represents the third component of the Muguga Cocktail [8]. Comparison of these genomes aims to provide insight into antigen gene variant combinations that render this vaccine cocktail particularly effective in the field, as well as providing baseline data on the component stocks. Additionally, the data generated can be used to develop single nucleotide polymorphism panels for high resolution monitoring of vaccine composition and of breakthrough infections.

## Results

### Whole genome sequencing data

The three isolates included in this study were sequenced on two different sequencing platforms, resulting in data with different read lengths and depth of coverage (Additional file 1: Table S1). For Serengeti-transformed and Muguga2, that were sequenced using 454 technology, 98.8 % and 98.1 % of the reads obtained aligned to the reference *T. parva* Muguga genome, respectively. For Serengeti-transformed, the mean read length was 541 bp, and the median was 549 bp, and for Muguga2, the mean was 470 bp and median was 480 bp. The total number of mapped bases corresponds to a theoretical sequence coverage of 58X for Serengeti-transformed and 29X for Muguga2.

The whole genome sequence data for the Kiambu5 stock was generated using Ion Torrent technology. Eighty three percent of all sequencing wells were used. Quality control resulted in the elimination of 30 % of reads, which were either primer dimers, or of low quality as determined by standard Ion Torrent filters. A total of > 3.6 million reads passed quality control checks, and these exhibited a mean length of 225 bp, and a median length of 209 bp. Of these, 94 % mapped to the reference *T. parva* Muguga sequence, resulting in theoretical sequence coverage of 39X for the Kiambu5 genome.

### DNA sequence polymorphism and genetic relationship among the components of the *T. parva* Muguga Cocktail

**Fig. 1:**
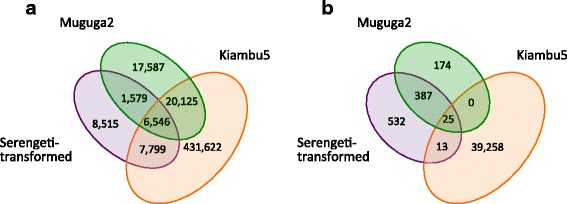
Single Nucleotide Polymorphisms (SNP) in each of the Muguga Cocktail component *T. parva* stocks relative to the reference *T. parva* Muguga isolate. **a**) Number of base pairs in the reference *T. parva* Muguga genome assembly with no read coverage from each of the three isolates. SNPs cannot be identified in this fraction of the genome, respectively 0.55 % (45,837 bp), 0.29 % (24,439 bp) and 5.61 % (466,092 bp) of the genome, in the comparisons with Muguga clone 2, Serengeti-transformed and Kiambu5. **b**) Identified SNPs unique to, and shared among, Muguga clone 2, Kiambu5, and Serengeti-transformed isolates

The level of genome sequence similarity between these three *T. parva* stocks included in the Muguga Cocktail, namely Muguga, Serengeti-transformed, and Kiambu5, is high but variable, as determined by SNP densities that vary between 0.10 SNPs/Kb for Serengeti-transformed to 4.70 SNPs/Kb for Kiambu5 relative to Muguga (Table 1). These SNP densities are likely an underestimation, since SNPs can only be identified in the regions of the reference genome to which sequence reads from the query isolate map. In fact, there was incomplete genome coverage from all isolates, with 0.55 %, 0.29 % and 5.61 % of the Muguga genome lacking read coverage from Muguga2, Serengeti-transformed and Kiambu5, respectively (Fig. 1).

**Table 1:** Number of SNPs identified between each *T. parva* stock in the Muguga Cocktail and the reference Muguga strain

Isolate	Total number of SNPs	Synonymous	Non-synonymous	Nonsense	Indels	Intron	Number of genes with non-syn SNPs
Serengeti-transformed	957	291	420	1	0	121	53
Kiambu5	39,296	20,086	8,587	16	748	7,615	1,708
Muguga2	586	206	264	1	0	48	52

Sequencing of Muguga2, a second clone derived from the Muguga stock, that is different from the clone used to generate the *T. parva* reference genome, is included primarily as a reference for internal variation within the stock. The results confirm that Muguga2 is nearly identical to the reference genome, with only 586 variants identified across the whole genome (Table 1). The Kiambu5 isolate is the most distinct of the three, with 39,296 SNPs relative to the *T. parva* Muguga reference. The majority of sequence variants between the Kiambu5 and the reference Muguga isolate, a subset of 27,732 SNPs, are shared with the KiambuZ464 clone that was sequenced previously by Hayashida and collaborators [10]. Unexpectedly, Serengeti-transformed appears to be remarkably similar to the Muguga reference, with only 957 sequence variants identified between the two isolates, 416 of which are shared with Muguga2 (Fig. 1). The SNPs found in each isolate were classified into synonymous, non-synonymous, intronic or intergenic based on the updated annotation of the reference genome assembly of the *T. parva* Muguga isolate (see Methods for details on re-annotation).

Despite the fact that these stocks were not cloned prior to cattle infection, no biallelic SNPs were detected in Serengeti-transformed, and only 18 of the 39,296 SNPs were scored as biallelic in Kiambu5, well within the error rate of Ion Torrent sequencing technology [11]. This result suggests that, in both isolates, only a single clone was detectable at the piroplasma stage of infection.

**Fig. 2:**
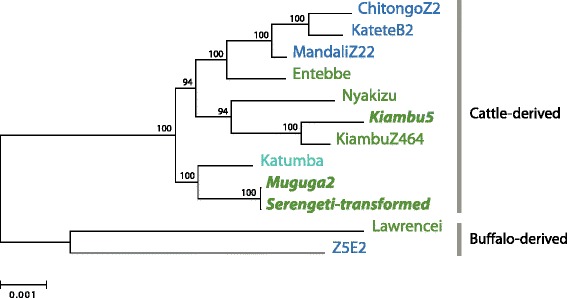
Phylogenetic relationships of twelve *T. parva* isolates. Genomic distances are measured in SNPs detected within the coding region of 200 randomly selected genes. Support was obtained from 500 bootstrap replicates. Represented isolates include *T.* parva stocks analyzed in this study, and the additional stocks described by Hayashida *et al.* in [10], namely the cattle-derived *T. parva* parasite stocks "*Entebbe*", "*Katumba*", "*Nyakizu*", "*ChitongoZ2*", "*KateteB2*", "*KiambuZ464*", and "*MandaliZ22*", as well as the African Cape buffalo-derived stocks "*Lawrencei*", and "*Z5E5*". Stocks are labeled according to country of origin: northern end of the distribution -Kenya, Uganda and Rwanda (dark green); north-central region –Tanzania (teal); central region –Zambia (blue). The three vaccine isolates are shown (bold-italics font)

We used the isolates sequenced in this study, as well as other cattle and buffalo strains previously sequenced by Hayashida et al. [10], to determine the relationship among the three constituents of the Muguga Cocktail in the context of the genomic information available for *T. parva* (Fig. 2). Our analysis shows the Kiambu5 stock clusters with the previously sequenced Kiambu clone, KiambuZ464, as would be expected. However, the Serengeti-transformed stock did not cluster with other buffalo-derived strains, and instead appears to be extremely similar to the Muguga reference strain. This indicates that the Serengeti-transformed stock currently used in the Muguga Cocktail vaccine most likely is not the original buffalo-derived stock.

### Whole genome differences between Serengeti-transformed and Muguga

The high similarity between these two isolates allows the detailed scrutiny of the mutations most likely to be of phenotypic relevance. Sequence reads from the Serengeti-transformed isolate mapped with 100 % coverage to 4063 of 4084 protein-coding genes in the reference *T. parva* Muguga isolate, a reflection of the negligible nucleotide sequence divergence between the two isolates. Of the 21 genes with incomplete read coverage, ten have less than 90 % sequence coverage. All these 21 genes are members of multigene families, including *Tpr* (*Theileria parva* repeat), sub-telomeric variable secreted protein (SVSP)-like and *Sfi*I-like genes, and the lack of read mapping is likely the result of mapping ambiguity. Of the 957 variants between Serengeti-transformed and Muguga, 291 are synonymous, 420 are non-synonymous, and one is a nonsense mutation. From this set of variants, 416 are shared between the Serengeti-transformed and Muguga2 stocks, including the nonsense mutation. This nonsense mutation is in the 4th amino acid in the protein encoded by TpMuguga_03g00616 (TP03_0616 in the original annotation; see Methods for details regarding new nomenclature), a locus that is part of the hyper-variable *Tpr* region, in chromosome 3 of *T. parva* Muguga. The *Tpr* locus consists of a series of tandemly arrayed, rapidly evolving genes [12–14] and in *T. parva* Muguga has five predicted transmembrane domains, suggesting that the protein encoded is membrane-associated. The presence of the nonsense mutation implies that not all proteins encoded in the *Tpr* locus are functional in all strains of *T. parva*. Alternatively, one of several downstream in-frame methionine-encoding codons might function as the true start codon.

**Fig. 3:**
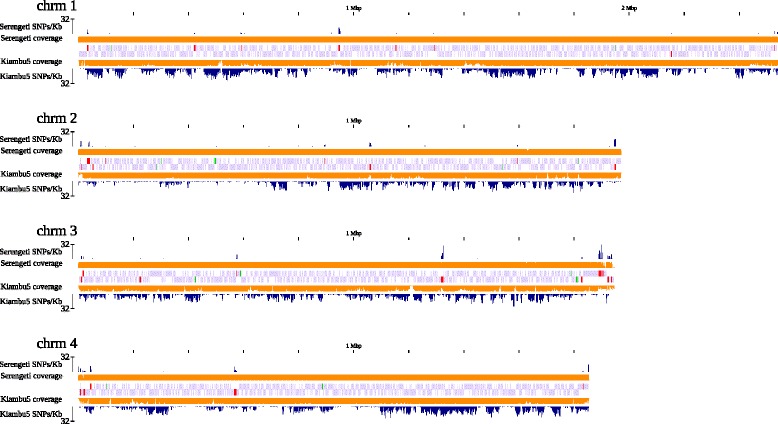
Distribution of variants in the Serengeti-transformed and Kiambu5 isolates relative to the genome of the *T. parva* Muguga reference isolate. For each of the four *T. parva* Muguga chromosomes the location of the protein coding genes in shown (purple), with genes encoded in the forward and reverse strands in the top and bottom central tracks, respectively. Genes with non-synonymous differences between Serengeti and Muguga are shown in red. Known antigens are shown in green. Variants were estimated in 2Kb-long, non-overlapping windows; for each of the isolates, the proportion of the window with read coverage (orange; min-max = 0-100 % coverage) is displayed, as is the number of SNPs per window (blue; min-max = 0-32 SNPs/Kb)

In total, 61 protein-coding sequences (CDSs) have changes relative to Muguga, with 53 having non-synonymous variants (Additional file 2: Table S2). These CDSs are clustered in a few specific genomic locations, in particular close to the telomeres (Fig. 3). A total of 14 genes encode proteins with well-characterized function, 13 of which contain non-synonymous SNPs (Table 2).

**Table 2:** Loci encoding well-characterized peptide products, which contain SNPs between the Serengeti-transformed stock and reference Muguga strain. Variants were also found in 47 hypothetical proteins (Additional file 2: Table S2)

Locus Tag /2005	Locus Tag ID^a^	Product Name	TM Domains^b^	Protein Localization / Confidence^c^	Serengeti-transformed SNPs^d^		Kiambu5 SNPs^e^	
					Syn	Nsyn	Syn	Nsyn
TP01_0011	TpMuguga_01g00011^f^	ABC transporter	11	_ / 2	9	11	≥66^f^	≥14^f^
TP02_0016	TpMuguga_02g00016^f^	ABC transporter	13	_ / 2	2	2	≥21^f^	≥7^f^
TP02_0017	TpMuguga_02g00017^f^	ABC transporter	12	_ / 3	19	17	≥11^f^	≥8^f^
TP02_0940	TpMuguga_02g00940^f^	ABC transporter	12	_ / 1	1		≥0^f^	≥0^f^
TP02_0951	TpMuguga_02g00951^f^	ABC transporter	11	_ / 1	44	37	≥9^f^	≥2^f^
TP03_0007	TpMuguga_03g00007^f^	ABC transporter	11	_ / 1	7	4	≥11^f^	≥9^f^
TP03_0864	TpMuguga_03g00864	ABC transporter	13	_ / 2	5	3	10	6
TP04_0021	TpMuguga_04g00021^f^	ABC transporter	13	_ / 1	10	24	≥43^f^	≥23^f^
TP02_0449	TpMuguga_02g00449	DNA replication factor CDT1 like	0	_ / 2	4			1
TP02_0448	TpMuguga_02g00448	F-box-like/WD repeat-containing protein TBL1XR1	0	_ / 1		1		1
TP03_0280	TpMuguga_03g00280	Papain family cysteine protease	1	_ / 2	12	7	1	1
TP04_0278	TpMuguga_04g00278	Translation initiation factor IF-2	1	S / 2	4	8		5
TP04_0279	TpMuguga_04g00279	Translation initiation factor IF-2	1	S / 2	1	3		
TP04_0280	TpMuguga_04g00280^f^	Translation initiation factor IF-2	1	S / 1	1	3	≥2^f^	≥2^f^

^a^Locus Tag Identifiers according to the updated whole genome gene structural re-annotation

^b^Number of transmembrane domains

^c^Protein localization predictions based on TargetP: C = chloroplast; M = mitochondrion; S = Secretory pathway; _ = Any other location. Prediction confidence: 1 = max; 5 = min

^d^SNPs between the Serengeti-transformed isolate and the reference Muguga isolate. Both synonymous (Syn) and non-synonymous (Nsyn) SNP counts are shown

^e^SNPs between Kiambu5 and the reference Muguga isolate

^f^This genes coding sequence(s) had at least one position with <10x mapping coverage in the Kiambu5 stock, and thus there is likely more variation in the actual sequence

Notably, seven of these genes with non-synonymous SNPs encode ABC-transporters, including the previously characterized TpABC2 (TpMuguga_03g00864) [15], with the remaining six comprising three translation initiation factor IF-2 genes, an F-box-like/WD repeat-containing protein TBL1XR1, a DNA replication factor CDT1-like, and a papain family cysteine protease. The 61 variant genes were evaluated for the presence of trans-membrane and GPI-anchored motifs and for predicted product localization. While no GPI-anchored peptides were identified, all the eight ABC-transporters had 11-13 trans-membrane domains, while the three translation initiation factor IF-2 genes and the papain family cysteine protease all have a single putative trans-membrane domain.

The ABC transporter gene family is characteristically located in the genomic region that separates sub-telomeric repeats families from the core of the chromosomes where most single copy genes are located [13]. The polymorphic TpABC2 locus and its homologs were evaluated further to determine the location of the variant residues. The trans-membrane regions showed non-synonymous SNP densities from 0.0 – 1.50 %, while non-synonymous SNP densities for intra- and extra-cellular regions were generally 0.0-1.68 %, and 0.0-1.60 % respectively. Notably though, TpMuguga_04_00021 had a non-synonymous SNP density of 7.3 % in its predicted intracellular regions.

Papain cysteine proteases are of particular interest in apicomplexan vaccinology. In *T. parva* they are thought to facilitate lymphocyte invasion [16]. Papain family cysteine proteases have been considered as potential vaccine targets in the related apicomplexan *Plasmodium falciparum* [17], and the previously described *T. parva* antigen Tp8, in chromosome 2 (TpMuguga_02g00140), is a papain family cysteine protease. The polymorphic papain cysteine protease identified here (TpMuguga_03g00280) is located in chromosome 3, and its antigenic potential warrants further investigation.

Finally, the translation initiation and replication factors, as well as the F-box-like protein, are all involved in transcription regulation. Their presence among this small set of highly variable proteins is unexpected but not entirely surprising, since one of the previously identified antigens, Tp5, is a eukaryotic initiation factor EIF-1A.

Parasite multigene families, often encoded in subtelomeric regions, play a fundamental role in pathogenesis of eukaryotic parasites, most notably in evasion of the host immune system, and as such they tend to be quite variable [18–21]. In *T. annulata*, the Tash1/SuAT1 family proteins have a potential role in host cell transformation and possibly in host cell immortalization [22] and there are homologues of these genes in *T. parva* that have not yet been tested functionally. The SVSP family is a subtelomeric gene family with a highly complex expression pattern [23]. Therefore, it is not surprising that many of the 47 genes with nucleotide differences between Muguga and Serengeti-transformed, which do not encode a well-characterized protein, belong to multigene families (Additional file 2: Table S2). Of these, 17 are members of the hyper variable Tpr (*Theileria parva* repeat) region. All these genes have between 1 and 11 transmembrane regions, and some contain putative domains. In addition, nine genes show high similarity (either full length or partial) to the *Theileria annulata* SfiI family. The SfiI family is a sub-telomeric gene family characterized by the presence of an *Sfi*I restriction site. Twelve of the 47 uncharacterized proteins have strong sequence similarity to the *T. parva* sub-telomeric variable secreted protein (SVSP) family, including two full length proteins as well as ten with similarity only over a terminal end of the protein, including four of the SfiI family proteins mentioned above, as well with six other hypothetical proteins. Finally, one gene had a predicted zinc finger and BTB domain-containing at the amino terminus, one was very similar to a *T. annulata* cysteine repeat modular protein 2, one to the *T. annulata Tash1* gene, one to *Babesia bigemina Vam6/Vps39-like* gene, and finally eight genes had no non-hypothetical BLASTx hits, although some contained conserved domains.

### Whole genome sequence diversity between Kiambu5 and Muguga

A total of 39,296 variants were identified between Kiambu5 and the reference *T. parva* Muguga isolate, affecting the coding region of 2,233 CDSs. Of these variants, 8,587 are non-synonymous mutations, which fall in 1,708 protein-coding genes (Table 1, Additional file 3: Table S3). Two patterns are markedly different from what was determined in the Serengeti-transformed vs. Muguga comparison. First, instead of primarily restricted to subtelomeric regions, the non-synonymous mutations are distributed throughout the genome (Fig. 3). Secondly, for a large number of genes in the *T. parva* Muguga reference genome, coverage with reads sequenced from the Kiambu5 genome is either partial (804) or absent (16), a result of the considerable sequence divergence observed between the two isolates.

Among all sequence variants found, 13 SNPs are exclusively shared with Serengeti-transformed, 25 with both Serengeti-transformed and Muguga2, and no SNPs are shared between Kiambu5 and Muguga2 to the exclusion of Serengeti-transformed (Fig. 1). Of the 13 SNPs shared between Kiambu5 and Serengeti-transformed, five are intergenic, four are located in TpMuguga_04g00278, two in TpMuguga_01g02945, and one each in TpMuguga_02g00449 and TpMuguga_02g00020. All these genes encode hypothetical proteins. Using blastx against the NCBI non-redundant gene database reveals a conserved Cdt1_m superfamily domain in TpMuguga_04g00278, and a translation factor II like superfamily domain in TpMuguga_02g00020.

Of the 61 CDSs that differed between Serengeti-transformed and the Muguga reference, 30 also have detectable differences in Kiambu5 (Additional file 2: Table S2; Table 2). Of these, three CDSs have more than 10 non-synonymous changes relative to Muguga, namely two of the ABC transporters (TpMuguga_01g00011 and TpMuguga_04g00021) and one hypothetical protein with sequence similarity to the *Sfi*I family (TpMuguga_03g00114). When compared to the Muguga reference, the latter has only a single non-synonymous SNP in the Serengeti-transformed stock but 21 non-synonymous polymorphisms in Kiambu5, while the two ABC transporters are among the 15 genes with a large number of non-synonymous SNPs between Serengeti-transformed and the Muguga reference genome. The accurate number of SNPs in many of these 61 genes could not be estimated from read mapping between Muguga and Kiambu5 due to incomplete read coverage over the length of each gene. The incomplete read coverage suggests that there are additional differences between the two isolates, and at sufficient density, to preclude Kiambu5 reads to map to the sequence of their Muguga homologs (i.e., >2 % sequence divergence), although other explanations exist (see Discussion). Therefore, these are in fact highly polymorphic genes.

### Nucleotide sequence variation in known *T. parva* antigens

To date, fourteen *T. parva* genes have been identified as antigens recognized by antibodies or T cells (Table 3). These antigens have in some cases been demonstrated experimentally to induce an immune response in the cattle host, but no single antigen, or evaluated combination of antigens, confers immunity to both *T. parva* experimental sporozoite needle infection and tick field challenge after delivery as recombinant subunit vaccines [24, 25]. It is, however, possible that one or more of these antigens contribute to protective immunity when presented in the context of challenge with the live parasite.

**Table 3:** List of known *T. parva* antigens described in literature. No variants were found for Serengeti-transformed in any of these loci, so only Kiambu5 nucleotide sequence variants are reported

Antigen	Locus Tag /2005	Locus Tag /2014	Description	Protein Localization/ Confidence	Domains	Kiambu5 variants			Ref.
						Syn	Non-syn	Intron	
Tp2	TP01_0056	TpMuguga_01g00056	CD8+ T cell target antigen	0 / S	-	2	2		[25]
gp34	TP01_0939	TpMuguga_01g00939	Hypothetical protein TP01_0939	1 / S	1 TM, 1 GPI	16	6		[47]
p32	TP01_1056	TpMuguga_01g01056	Antibody target antigen 32 kDa surface protein	0 / S	1 GPI	3	8		[25]
Tp8	TP02_0140	TpMuguga_02g00140	CD8+ T cell target cysteine proteinase	1 / _	1 TM	0	1		[25]
X88	TP02_0148	TpMuguga_02g00148	T cell target heat shock protein 70	0 / _	-	16	0		[48]
Tp7	TP02_0244	TpMuguga_02g00244	CD8+ T cell target antigen heat shock protein 90	0 / _	-	0	0		[25]
Tp5	TP02_0767	TpMuguga_02g00767	CD8 + T cell target antigen translation initiation factor eIF-1A	0 / _	-	1	0	6	[25]
Tp9	TP02_0895	TpMuguga_02g00895	CD8+ T-cell target antigen Tp9	0 / S	-	?	?		[49]
Tp4	TP03_0210	TpMuguga_03g00210	CD8 + T cell target antigen T-complex protein 1 subunit eta	0 / _	-	22	1	10	[25]
p67	TP03_0287	TpMuguga_03g00287	Antibody target antigen p67 sporozoite surface protein	1 / S	1 TM, 1 GPI	0	0		[24]
Tp1	TP03_0849	TpMuguga_03g00849	CD8 + T cell target antigen apical membrane antigen 1	1 / S	1 TM	1			[25]
p150	TP03_0861	TpMuguga_03g00861	Antibody target antigen p150 microsphere protein	0 / S	-	0	0		[50]
PIM	TP04_0051	TpMuguga_04g00051	Antibody target antigen polymorphic immunodominant molecule (PIM protein)	3 / S	3 TM	0	0		[51]
p104	TP04_0437	TpMuguga_04g00437	Antibody target antigen 104 kDa rhoptry protein	1 / S	1 TM, 1 GPI	3	13	2	[52]

Interestingly, Serengeti-transformed is identical to the Muguga reference genome in all of the currently described antigens. Kiambu5 shows a number of variations within this set of genes, with 65 synonymous and 31 non-synonymous SNPs detected. In total, six of the known antigens have non-synonymous changes, namely the CD8 T cell target schizont antigen Tp2, Tp4, Tp8, and also gp34, p32, and p104. In addition, three proteins, namely the p32, Tp4, and PIM antigens all had segments with no coverage, which precluded reliable SNP identification in these regions, and suggested substantial differences between the two isolates. Finally, no Kiambu5 reads mapped to the Muguga Tp9 locus, a highly variable antigen, suggesting the presence of widespread nucleotide differences over the entirety of the locus between these two isolates. The sequence of PIM is known for Kiambu5, and very different to that of Serengeti-transformed and Muguga, which are identical [26].

Most of the genes have no trans-membrane motifs and none has more than four, which indicates that none of these are likely to be membrane constituents. Tp4, Tp5 and p150 all contain GPI-anchoring motifs. Most of these are targeted to the secretory pathway with high reliability, confirming the widespread assumption that secreted proteins are likely to be targeted by the host immune system during the schizont stage. Only half of these genes contain non-synonymous polymorphism, a frequency that is not significantly different from that found for the whole genome (*P* = 0.2386; one-tailed Fisher’s exact test) and suggests that a high level of polymorphism is not a necessary property of antigens. However, some of these antigens are indeed extraordinarily polymorphic, such as p32, p104 and gp34 with respectively 8, 13 and 6 non-synonymous polymorphisms relative to Muguga, and the Tp9 antigen apparently so distinct that no reads map across isolates within the default read mapping stringency of BWA. This conclusion is supported by the fact that this gene is highly polymorphic, with average pairwise difference between alleles well above 10 % (Silva, unpublished).

### Identification of additional putative antigens

**Fig. 4:**
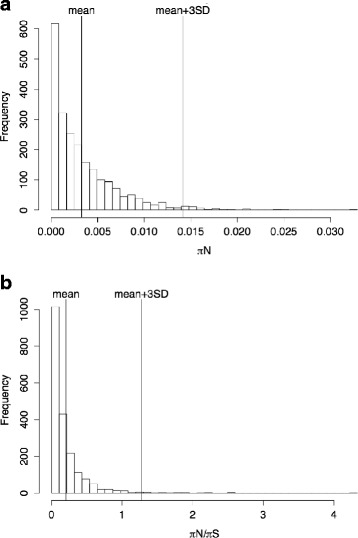
Distribution of πN and πN/πS for all genes in a comparison between two strains of *T. parva*. Histograms of Histograms of (**a**) the distribution of non-synonymous polymorphism (πN) and (**b**) the ratio of non-synonymous to synonymous polymorphism in a comparison between the sequence of Kiambu5 and the reference Muguga isolate. Show are the mean, as well as the mean + 3*SD which is the cut-off used for Additional file 5: Table S5

The interaction between antigens and the host immune system can lead to positive selection, either due to the increased fitness of non-synonymous polymorphisms or due to frequency-dependent selection, leading to an increase in *π*_N_ and in *π*_N_/*π*_S_ within genes encoding antigens relative to other genes. Hence, signatures of positive selection are often used to identify potential antigens [27–30]. Accordingly, we searched for genes potentially evolving under positive selection using the comparison between the Muguga reference and Kiambu5 alleles according to three different criteria: *i*) high *π*_N_, a measure of the proportion of non-synonymous sites that differ between the alleles, (*ii*) high *π*_N_/*π*_S_, a measure of the rate of amino-acid changing mutations relative to the rate of silent mutations, and (*iii*) a less well-defined criterion, namely Muguga single copy gene for which a high proportion of the length has no coverage from Kiambu5 reads, suggesting that the level of sequence divergence between the two isolates is quite high, with a resulting SNP density that prevented successful read alignment. In the comparison between the Muguga reference and Kiambu5, *π*_N_ varied between 0 – 3.29 %, with a modal value of 0.16 % (Fig. 4a). The genes with the highest 5 % *π*_N_ values, and that encode a non-hypothetical protein, are listed in Table 4. The values of *π*_N_/*π*_S_ ranged between 0 and 4.33, for genes with a valid *π*_N_/*π*_S_ ratio, and the distribution has a modal value of 0.11 (Fig. 4b).

**Table 4:** Loci encoding well-characterized peptide products, with the highest 5 % πN value between the Kiambu5 stock and the Muguga reference sequence. Variants in hypothetical proteins, and those not in the highest 5 % πN can be found in Additional file 3: Table S3

Locus Tag / 2005	Locus Tag ID^a^	Product Name	Domains	Protein Localization/ Confidence^c^	πN^d^	πN/πS^d^	Syn^d^	Non-syn^d^
TP01_0066	TpMuguga_01g00066	50S ribosomal protein L33	-	S/ 1	0.013	0.4313	3	3
TP01_0248	TpMuguga_01g00248	tRNAHis guanylyltransferase	1 TM^**b**^	_/ 1	0.0148	0.4679	8	10
TP01_0272, TP01_0273	TpMuguga_01g00272	Kinase binding protein CGI-121	-	_/ 1	0.0161	0.3261	7	8
TP01_0374	TpMuguga_01g00374	DEAD/DEAH box helicase	-	_/ 2	0.0131	0.3503	13	15
TP01_0405	TpMuguga_01g00405	Asparagine synthetase domain-containing protein C4F6.11c	-	_/ 1	0.0121	0.4375	14	15
**TP01_1056**	**TpMuguga_01g01056**	**Merozoite Antigen**	1 GPI	S/ 2	0.0125	1.1773	3	8
TP01_1165	TpMuguga_01g01165	ATP-dependent RNA helicase SUV3 homolog mitochondrial	-	M/ 5	0.015	0.3049	25	23
TP01_1173, TP01_1174	TpMuguga_01g01174	Ribonuclease P protein subunit p29	-	_/ 3	0.0123	0.255	7	7
TP01_0275, TP01_0273, TP01_0274, TP01_0272	TpMuguga_01g02345	Phosphatidylinositol 4-phosphate 5-kinase 7	-	_/ 4	0.0142			3
TP01_1063	TpMuguga_01g02455	Uncharacterized protein C18H10.09	-	_/ 1	0.0132	0.66	2	4
TP02_0369	TpMuguga_02g00369	Dual specificity protein phosphatase 12	-	_/ 2	0.0142	0.3435	11	11
TP03_0010	TpMuguga_03g00010	Archease protein family (MTH1598/TM1083)	-	_/ 1	0.0143	0.4618	6	8
TP03_0595	TpMuguga_03g00595	Ydr279p protein family (RNase H2 complex component)	-	_/ 2	0.0187	0.5673	5	13
TP03_0601	TpMuguga_03g00601	CobW/HypB/UreG nucleotide-binding domain	-	_/ 3	0.0127	0.4562	9	10
TP03_0270	TpMuguga_03g02310	Protein CyaY	-	_/ 5	0.0117	0.2878	36	35
TP04_0705	TpMuguga_04g00705	Maf-like protein	-	_/ 3	0.0115	0.5954	5	6

The previously identified antigen p32 is marked in bold

^**a**^Locus Tag Identifiers according to the updated whole genome gene structural re-annotation

^**b**^Number of transmembrane domains

^**c**^Protein localization predictions based on TargetP: C = chloroplast; M = mitochondrion; S = Secretory pathway; _ = Any other location. Prediction confidence: 1 = max; 5 = min

^**d**^SNPs between Kiambu5 and the reference Muguga isolate

The lack of read mapping across specific regions of isolate genomes is almost certainly a reflection of low sequence similarity, and hence an indicator of rapid evolution rates (but see Discussion). A total of 16 Muguga genes (corresponding to 0.39 % of all protein-coding genes) completely lacked read coverage in Kiambu5, and 183 genes (4.48 %) had less than 10x mean coverage, preventing variant calling. Of these 199 (16 + 183), 163 are single copy genes. We identified the 100 genes with the lowest read mapping percentage (Additional file 4: Table S4). These include 31 novel genes, which were absent from the original annotation of the Muguga genome, and which are now part of the annotation being released in 2015 (http://jbrowse.igs.umaryland.edu/t_parva/). In total, these 100 include 69 hypothetical proteins, 20 genes lacking annotation, three contain conserved DUF529 domains of unknown function, three DNA-directed RNA polymerase subunit beta, and one of each ribose 5-phosphate isomerase A (phosphoriboisomerase A), TBC domain-containing protein kinase-like protein, DNA-directed RNA polymerase subunit gamma, elongation factor Tu GTP binding domain, and H/ACA ribonucleoprotein complex subunit 3. Five of these 100 genes also had modifications between Serengeti-transformed and Muguga, all hypothetical proteins (Additional file 2: Table S2). One of these (TpMuguga_04g00001) has paralogs in the genome, the remaining four being TpMuguga_01g00288, TpMuguga_01g00464, TpMuguga_02g00526, and TpMuguga_02g00527, which are all close to the telomeric region of their respective chromosomes.

Finally, we compiled a group of genes with a *π*_N_ or *π*_N_/*π*_S_ value greater than three standard deviations above the mean value of each statistic, comparable to the selection criteria used by Hayashida [10], and supplemented with the genes selected in this study according to any of the criteria listed above that were also detected by the Hayashida study (Additional file 5: Table S5). The two studies obtained slightly different distributions for *π*_N_/*π*_S_ values, which averaged 0.21 ± 0.36SD in the present study, compared to the study of Hayashida and colleagues [10] with average of 0.49 ± 0.31SD; the difference is not surprising given that both studies are based on a small number of parasite stocks, which differ between the studies, and on different methodologies, both in terms of read length, as well as stringency of read mapping and SNP calling. Interestingly, despite the use of different isolates and different selection criteria, of the 262 genes prioritized by Hayashida and collaborators, and the 175 prioritized in this study, 81 genes are present in both sets. The 81 genes identified in both studies include many of the known antigens, and is likely to represent a subset of particular interest in terms of antigenic potential (Additional file 5: Table S5).

## Discussion

### The importance of the Serengeti-transformed and Kiambu5 isolates

The Muguga Cocktail vaccine is composed of three different isolates, each of which is thought to contribute to the efficacy of ITM. With the goal of identifying unique features that might be provided by each strain we sequenced and analyzed the components of this vaccine cocktail. A striking result from this study is the remarkable similarity between the Serengeti-transformed genome and that of the Muguga reference strain. When compared to Muguga, Serengeti-transformed shows only roughly 1.5 times the number of SNPs found in the Muguga2 clone, which is a variant derived from the Muguga stock. This represents 41 times fewer SNPs than were identified between Kiambu5 and Muguga, and almost 100 times less than the previously published Marikebuni and Uganda stocks, when compared to *T. parva* Muguga [8]. The Serengeti-transformed stock was originally a component of a buffalo isolate that was experimentally adapted to cattle through tick passage [3]. Given the high level of sequence divergence observed between *T. parva* isolates from cattle and the few available buffalo-derived isolates [10], it is very surprising that the original Serengeti-transformed stock would be so similar to the cattle-derived Muguga stock. Three possible scenarios can explain this observation. It is conceivable that the Serengeti-transformed stock is indeed a buffalo isolate which is nevertheless unusually closely related to the Muguga reference isolate, making the 52 annotated CDS with non-synonymous SNPs between the Serengeti-transformed and Muguga stocks extremely interesting from the point of view of their potential to confer protective efficacy. Also conceivable is the possibility that the Serengeti-transformed stock was a mixed stock containing both more “typical” buffalo-derived strains as well as a strain similar to cattle-derived isolates and very similar to Muguga, with the latter being preferentially selected during recurrent passage. Finally, the currently used Serengeti-transformed stock that is contained within the Muguga Cocktail represents a historical contamination with a *T. parva* Muguga clone. The fact that the Serengeti-transformed strain analyzed here shares 416 SNPs with Muguga2 (about 71 % of all Muguga2/Muguga SNPs) indicates that the two are likely to share a very recent common ancestor, and favors this last scenario.

Regardless of its relationship with Muguga stock, the Serengeti-transformed strain analyzed here is in fact the component currently used under that name in the Muguga Cocktail. This vaccine formulation is effective in the field, but our observations raise the question of whether the inclusion of this Serengeti-transformed strain is necessary for the Muguga Cocktail to be protective, or if instead the preparation of the Muguga Cocktail could be streamlined by eliminating this strain. In fact, the need to re-investigate the contribution of the Serengeti-transformed strain to the Muguga Cocktail was first discussed as early as 2001 [4]. Interestingly, however, despite the surprisingly high sequence similarity between Muguga and Serengeti-transformed, there are several proteins that differ considerably between those two stocks. These highly variably loci include members of the *Tpr* repeat family, ABC-transporters, a papain-family cysteine protease, and proteins which show sequence similarity to the SVSP and SfiI families, predicted to be involved in parasite host interaction. Sub-telomeric gene families are often involved in host-pathogen interactions, making these genes of particular interest.

Finally, whether or not the re-introduction of a more divergent, buffalo-derived *T. parva* stock could improve further the protective performance of the Muguga Cocktail under different field conditions will require further studies. One possible constraint is the difficulty to propagate buffalo-derived strains in ticks and cattle to generate sufficient quantities for stabilate production.

Incomplete coverage with mapped reads can result from several factors including (*i*) heterogeneity in cloning and/or sequencing efficiency of different genomic regions (say, those differing in GC content), (*ii*) the fact that whole genome shotgun sequencing coverage follows a Poisson distribution, with some regions, by chance, being sequenced at much lower level that others, and resulting in some regions with no coverage at all in most genomes sequenced to a reasonable depth of coverage, (*iii*) ambiguity in mapping location (e.g., reads that match equally well to multiple locations in the genome) or (*iv*) high degree of sequence divergence between sequence reads and the target genome. The difference in coverage between Muguga2 and Serengeti-transformed likely results from the difference in data volume (Additional file 1: Table S1) coupled with the random distribution of reads described in *(ii)* above. The fact that many of the genes with incomplete or no read coverage in the comparison between Muguga and Kiambu5 are known to be of average GC content and high polymorphism suggest that, for the most part, factor (*iv*) above is the main reason for the incomplete read coverage observed for Kiambu5. The comparison between Muguga and Kiambu5 clearly highlights the limitations of read mapping-based characterization of gene evolution rates. In particular, the precise quantification of genetic differences between divergent isolates is restricted to genes with low polymorphism. Reliable estimates of sequence polymorphism for rapidly evolving genes, and in-depth characterization of the selective pressures governing their evolution, will require *de novo* genome assemblies from which complete gene sequences can be extracted and analyzed.

### Implications of the sequence data for ITM vaccine quality control

Previous studies of the genetic composition of stocks and stabilates comprising the Muguga Cocktail utilized Southern blotting with multicopy probes [4], nucleotide sequencing and a panel of mini- and micro-satellite markers [31]. Application of these approaches [4, 26, 32] also revealed close similarity between Muguga and Serengeti-transformed including the sequence of the gene encoding the polymorphic immunodominant molecule (PIM). These two studies [26, 32] additionally revealed diversity within DNA prepared directly from the sporozoite stabilates. A more recent study using only five satellite markers [33], revealed even more heterogeneity within the DNA of Muguga and Serengeti-transformed stabilates, while Kiambu5 appeared to be clonal with these markers. The stocks that were used for infection of animals were not cloned, and these stabilate genotyping studies do, in fact, suggest that multiple clones could have been present in the initial sporozoite inoculum [32]. However, our analyses reveal the presence of a single genotype in both the Serengeti-transformed and Kiambu5 DNA samples. Taken together these observations suggest that, upon infection, only one of possibly multiple clones present will expand to become the predominant and only detectable parasite in the infection. The phenomenon of selective amplification of a predominant clonotype on passage through cattle and ticks has been observed previously. For example, when the Marikebuni stock was used to infect cattle, 48 clonal genotypes were reduced to 18 in a single passage, and 75 % of these were derived from one highly inbred genotype [34]. The study by Patel and collaborators [33] did not reveal the very extensive genetic divergence between Serengeti-transformed/Muguga and Kiambu5, perhaps because of the markers used, which were selected from a pool of 31 VNTR (variable number of tandem repeat) markers that were strongly biased towards those that detect diversity within the *T. parva* Muguga stock. The higher information content and value of SNP-based, genome-wide genotyping is clearly illustrated by the discrimination of Muguga and Serengeti-transformed at specific loci, particularly in the ATP binding cassette transporter family, despite their overall similarity.

The sub-telomeric SVSP gene family members are predicted to be part of the schizont secretome of *T. annulata* and *T. parva* that has expanded selectively when compared to the non-transforming *T. orientalis* genome [10]. Sequences of SVSP gene family members differ between all isolates compared here, giving each isolate a unique SVSP genomic signature. Investigation of cellular immune responses in ITM-vaccinated cattle using peripheral blood mononuclear cells stimulated by sets of overlapping synthetic peptides covering these variant SVSP is well justified based on the data presented in this study.

The close dependency on the Muguga reference genome poses significant challenges to the identification of novel features in each of the components of the Muguga Cocktail. As discussed above in terms of read mapping, genetic differences between the isolates can only be identified if they are sparsely distributed and located close to conserved regions that can anchor the mapped reads. In addition, the only genes analyzed were those present in the reference Muguga isolate, and so there are currently no features known to be unique to the Kiambu5 or Serengeti-transformed genomes, such as the presence of previously unknown genes or genomic rearrangements. While the IonTorrent 316 sequencing chip produced good quality data, it did not allow the generation of a high quality *de novo* genome assembly. Further sequencing using paired-end or long-read data will be needed to identify genomic rearrangements, sequences that are unique to specific stocks, and perform analyses of the full complement of rapidly evolving genes.

### Relevance to development of other whole-organism vaccines

Recently, there has been renewed enthusiasm for the prospect of the development of a effective whole live *Plasmodium falciparum* sporozoite vaccine, based on the encouraging outcome of a number of phase I clinical trials [35]. The possibility of manufacturing cryopreserved, metabolically active, live *P. falciparum* sporozoites (PfSPZ) suitable for clinical application has been a milestone for this vaccine development approach [36]. The *T. parva* Muguga Cocktail is composed of three isolates, two of which are nearly identical, and which together represent most likely only a small amount of the genetic variation circulating in the field (Fig. 2). The fact that this preparation is able to confer high levels of protection against highly diverse *T. parva* cattle-transmissible field isolates, and that population variation in *P. falciparum* is much lower than that is observed in *T. parva* [10, 37], is highly supportive of a similar initiative in malaria.

## Conclusions

We sequenced the whole genomes of three *T. parva* component stocks (Muguga Cocktail) that form the basis of the ITM procedure for control of East Coast fever in cattle in East Africa. The Serengeti-transformed isolate currently used in this cocktail, even though originally isolated from the main wildlife host, African Cape buffalo (Syncerus caffer), is very similar to the cattle-derived stock from which the *T. parva* Muguga reference isolate was cloned. All non-synonymous SNPs were found in only 53 genes, mostly sub-telomeric loci and/or genes predicted to encode antigenic proteins. The Kiambu5 stock is much more divergent, containing approximately 40,000 single nucleotide differences relative to Muguga, including >8,500 amino acid-changing mutations that affect 42.5 % of the predicted proteins. Importantly, the genetic content and variation of Kiambu5, Muguga and Serengeti-transformed represent only a small proportion of the genetic variation of cattle-derived *T. parva* isolates circulating in the field. Our results demonstrate that whole organism-based, live vaccines against highly polymorphic apicomplexan parasites can be highly effective. The Muguga cocktail does not, however, cross-protect against all buffalo-derived *T. parva* in Eastern Africa [38]. Genome-wide studies that focus on genetic differences between cattle- and buffalo-derived *T. parva* parasites will shed light on the genomic basis of vaccine evasion by the latter, and inform the design of more broadly protective vaccine preparations.

## Methods

### Biological samples

The three *T. parva* stocks that comprise the Muguga Cocktail version of ITM are compared in this study: Muguga, Kiambu5, and Serengeti-transformed. The origin of these stocks is as follows:

i. Muguga: the reference *T. parva* genome sequence [19] was derived from piroplasms, which were purified following infection of several cattle with the cloned parasite stabilate 3308 [39]. The *T. parva* Muguga clone 2 sequence was derived from piroplasms isolated from animal BM256 infected with a second Muguga cloned stabilate, 3968, that differs from the reference Muguga isolate in the organization of the hyper-variable *Tpr* locus, according to Southern blot data (R. Bishop, unpublished data).
ii. The Kiambu5 genome sequence was produced from purified piroplasm DNA generated from reference stabilate 4137, which itself is directly derived from the seed stabilate KV 68 that was used to produce the first bulk stabilate of the Muguga Cocktail vaccine, FAO1, by infection of animals and application of ticks [7].
iii. The Serengeti-transformed genome was produced from purified piroplasm DNA from an animal infected with the seed stabilate Serengeti-transformed 69 which represented the direct precursor present in the FAO1 ITM vaccine stabilate. The Serengeti-transformed stock was originally established from a buffalo isolate that was experimentally adapted to cattle through extensive tick passage [3].

### Ethics statement

The ILRI’s Institutional Animal Care and Use Committee (IACUC) was established in 1993 to ensure that international standards for animal care and use are followed in all ILRI research involving use of animals. ILRI has complied voluntarily with the UK's Animals (Scientific Procedures) Act 1986 (http://www.homeoffice.gov.uk/science-research/animal-research/) that contains guidelines and codes of practice for the housing and care of animals used in scientific procedures. The study reported here was carried out in strict accordance with the recommendations in the standard operating procedures of the ILRI IACUC and adequate consideration of the 3R's (Replacement of animal with non-animal techniques, Reduction in the number of animals used, and Refinement of techniques and procedures that reduce pain and distress). Generation of piroplasms for this work was done under IACUC-approved protocol with ILRI reference number 2011-07.

### Experimental infection of cattle, piroplasm purification and DNA extraction

For generation of piroplasms from Kiambu5, experiments were conducted using four Friesian calves each aged six months. Prior to infection, the cattle were screened using an indirect ELISA based on the recombinant PIM antigen and with a *T. parva-*specific p104 PCR assay to confirm that there was no prior exposure to *T. parva*. Each calf was infected with a standard dose (1:20 dilution) of a *T. parva* Kiambu5 reference stabilate derived from the Malawi 68 (KV68) vaccine seed stabilate. The diluted stabilate was inoculated subcutaneously below and in front of the left parotid lymph node. The animals were treated daily with a low dose (10 mg/kg) of oxytetracycline from day 10 post-infection. This procedure ensured that the clinical distress on the animal was reduced and also protected the animals against premature death prior to the development of piroplasm parasitaemia, which ranged between the relatively low levels of 0.98 and 1.62 %. During the course of infection, the animals were monitored for development of fever and presence of schizonts in blood and lymph node smears. Clinical reactions were recorded on standard ILRI animal record experimental data recording forms. Due to the typically low levels of piroplasm parasitaemia observed in cattle infected with *T. parva* Kiambu stocks, it was necessary that a large volume of blood be collected from all infected animals to purify sufficient amounts of piroplasms for DNA extraction. Exsanguination under general anesthesia was performed by carotid artery cannulation after the animals had developed a minimum piroplasm parasitaemia of 2 %. Venous blood was collected in Alsevers solution containing heparin at a concentration of 50 i.u/ml of blood and then centrifuged at 3500 rpm for 30 min to remove the serum and buffy coat layer. The cells were washed three times by re-suspending in fresh cold Alsevers solution and centrifuged at 3500 rpm for 30 min. Finally, the washed red blood cells were lysed in pre-warmed 1 mg/ml saponin at 37 °C for 30 min, then washed with Alsevers solution and centrifuged as before. The supernatant was collected using a trap bottle and centrifuged at 10,000 rpm for 30 min, then carefully discarded while retaining the piroplasm pellet, which was then washed three times in Alsevers and finally re-suspended in TEN- buffer (10 mM Tris-Cl;1 mM EDTA; 100 mM NaCl, pH 8.0). The purified piroplasms were then stored at -20 °C. Procedures for animal infection and purification of piroplasms from *T. parva* Serengeti-transformed and Muguga2 were similar, except that the administration of oxytetracycline was not used to prolong infection. Piroplasm samples for these two isolates were generated in or before 1999, in the context of previous studies [19], but the genome was not sequenced. Genomic DNA was subsequently prepared using standard phenol/chloroform and ethanol precipitation extraction procedure as described elsewhere [8].

### Genome sequencing strategy, read mapping, and variant calling

The Muguga2 DNA sample was sequenced at the Functional Genomics Centre Zurich, Switzerland, using a Roche 454 GS FLX sequencing platform with Titanium chemistry. A shotgun library and an 8 Kb mate-paired library were prepared and sequenced according to the manufacturer’s protocols. The Serengeti-transformed DNA sample was sequenced at ILRI using a similar sequencing strategy, except that the mate-paired library was prepared with 3 Kb inserts. *Theileria parva* stock Kiambu5 was sequenced at the Swedish National Genomics Infrastructure, in Uppsala, Sweden. Two samples, containing a total 2.2 μg genomic DNA were sheared to a target size of 300 bp, followed by two cycles of amplification. The samples were then pooled and the library finalized using the Ion OneTouch system with the Ion PGM template OT 300b kit. The library was sequenced on a 316 chip using the Ion PGM™ Sequencing 300 Kit on the Ion PGM™ system. The sequence data have been deposited with GenBank Short Read Archive database under BioProject accession number PRJNA276471.

The Kiambu5 reads were aligned against the Muguga reference strain (Genbank accession numbers: AAGK01000001- AAGK01000009) [19], using the Burrows Wheeler aligner (BWA) [40] with default parameters. Reads with a negative mapping score were removed. The Serengeti-transformed and Muguga2 reads were aligned with the GS Reference Mapper (454 Life Science), using the same Muguga reference strain. For each of the three isolates’ read dataset, the number of base pairs in the Muguga reference genome with no read coverage determined from the bam files, using a custom script.

Indels and SNPs for all isolates were identified using the Genome Analysis Toolkit (GATK) from the Broad Institute [41], and SNPs were filtered using SAMtools [42] according to the following filter: (DP < 12) || (QUAL < 50) || (SB > -0.10) || (MQ0 > =2 && (MQ0/(1.0*DP)) > 0.1), where DP is total read coverage depth, QUAL is quality, SB is strand bias, and MQ0 is the number of reads with mapping quality zero. SNPs were classified into synonymous, non-synonymous, nonsense, intronic, and indels based on the updated gene structural annotation of the entire reference *T. parva* Muguga genome assembly (http://jbrowse.igs.umaryland.edu/t_parva/; Silva, in preparation), using VCF_annotator (http://sourceforge.net/projects/vcfannotator/) and in-house python scripts towards the Muguga reference (GenBank accession numbers: AAGK01000001- AAGK01000009) [19]. The locus tag identifiers for the new annotation are very similar to those in the original annotation for genes with no or minor structural changes, such as the addition of UTRs or alterations of intron-exon boundaries (e.g., TP01_0056 simply becomes TpMuguga_01g00056). However, in the case of genes with a fundamentally difference structure, such as new genes, genes that have been split to result in two or more genes in the current annotation, or cases in which two or more original genes have been merged, then the gene numbering will be altered to start in the 2000’s (e.g., TpMuguga_01g02345 is located in chromosome 1, and has gene number 2345).

### Gene functional characterization and gene family identification

Additional information for loci of interest was obtained from publicly available sources. Transmembrane motifs were identified with TMHMM2.0 [23], and GPI-anchored motifs with GPI-SOM [43]. For selected genes for which the product was annotated as “hypothetical proteins” with our stringent functional assignment pipeline, we performed additional sequence similarity searches against the public databases GenBank (non-redundant proteins), SwissProt, and KEGG to determine if they belong to known protein families or contain known functional motifs.

In order to estimate the number and composition of multigene families in the *T. parva* genome, all genes were compared to the complete set of *T. parva* genes from the reference annotation using BLAST [44]. A gene was considered to have paralogs if it had at least one blastn hit with an expectation value ≤ 1e-3, covering at least 50 % of either the query gene or the target gene.

### Molecular evolution

In order to identify genes that encode rapidly evolving genes, and in particular those that may be evolving under positive selection, the variants identified in the Kiambu5 strain relative to the reference genome were used to generate Kiambu5 “pseudo-sequences”. These sequences correspond to the homologous sequence from the *T. parva* Muguga reference with the exception of the positions in which SNPs were identified. These “pseudo-sequences” are potentially conservative in their difference to the *T. parva* Muguga homologs, due to mapping stringency limitations and variant filtering. All coding sequences (CDSs) from the Muguga reference genome were aligned to the inferred Kiambu5 CDSs obtained from the pseudo-sequences, and *π*_N_/*π*_S_ ratios were calculated with the LPB93 algorithm of the yn00 program, as implemented in the PAML package [45], where *π*_N_ and *π*_S_ are, respectively, the rate of non-synonymous and of synonymous polymorphisms per site. All CDSs with an *π*_N_ > 0 were identified. The value of *π*_N_/*π*_S_ was estimated for those genes for which the statistic is valid (i.e., those for which *π*_S_ > 0).

### Genetic relationship between isolates

The relationship and relative genetic distances between isolates were visualized with a dendrogram created using the nucleotide sequences of 200 protein-coding genes, selected pseudo randomly using the Python2.7 random library (seed 1). The sequence of the selected genes in each isolate was inferred from the SNP calls, with “pseudo-sequences” generated as described above, and all genes were concatenated, creating pseudo-contigs. The pseudo-contigs were modified using a series of at least 20 N’s to separate genes and prevent frame-shifts. These pseudo-contigs were then aligned using clustalw2 [46], and a maximum-likelihood phylogeny was calculated with MEGA6, using default parameters which included the Tamura and Nei model of evolution. Clade support was estimated from 500 bootstrap replicates. The branch lengths in the dendrogram are only an approximation of the true genetic distance between isolates for several reasons, including that (*i*) the subset of ~5 % of all genes used to calculate the relationships may not be representative of the whole genome, (*ii*) gene sequences were inferred from SNPs identified through read mapping against the reference rather than from *de novo* genome assemblies, and (*iii*) since *T. parva* undergoes recombination, a bifurcating tree is most likely not a strictly correct depiction of the relationship between these genome sequences.

## Additional files

Additional file 1: Table S1. Sequencing data statistics for three *T. parva* stocks. (XLSX 49 kb) Additional file 2: Table S2. Comprehensive lists of variants identified in *T. parva* Serengeti-transformed and Kiambu5 stocks, relative to the reference *T. parva* Muguga isolate, respectively. (XLSX 65 kb) Additional file 3: Table S3. Comprehensive lists of variants identified in *T. parva* Serengeti-transformed and Kiambu5 stocks, relative to the reference *T. parva* Muguga isolate, respectively. (XLSX 71 kb) Additional file 4: Table S4. Shows the 100 genes in the Muguga reference genome sequence with the lowest average read mapping coverage from Kiambu5 whole genome shotgun sequencing reads. (XLSX 37 kb) Additional file 5: Table S5. Lists the genes selected by πN/πS > mean + 3*SD, πN > mean + 3*SD, and genes highlighted in both this study and by Hayashida *et al.* [9]. (XLSX 45 kb)

## Abbreviations

ECF: East coast fever
ITM: Infection and treatment method
SNP: Single-nucleotide polymorphism
